# A Case of Anti-neutrophil Cytoplasmic Antibody-Associated Vasculitis Presenting With Diffuse Alveolar Hemorrhage and Renal Sparing

**DOI:** 10.7759/cureus.45397

**Published:** 2023-09-17

**Authors:** Ahmed Alobaidi, Ahmed Albadry, Anne Murray, Irina Lytvak

**Affiliations:** 1 Department of Internal Medicine, Methodist Health System, Dallas, USA; 2 Faculty of Medicine, Charles University in Prague, Prague, CZE; 3 Clinical Research Institute, Methodist Health System, Dallas, USA; 4 Department of Pathology, Methodist Health System, Dallas, USA

**Keywords:** anca vasculitis with renal sparing, necrotizing vasculitis, anca associated vasculitis, anti-neutrophil cytoplasmic antibody, diffuse alveolar hemorrhage

## Abstract

Anti-neutrophil cytoplasmic antibody (ANCA)-associated vasculitis (AAV) is a necrotizing vasculitis disease that traditionally includes three variants classified based on their clinical and pathological appearance: microscopic polyangiitis (MPA), granulomatosis with polyangiitis, and eosinophilic granulomatosis with polyangiitis (alternatively, Churg-Strauss syndrome). The mainstay of AAV treatment is immunosuppressive treatments, which improve survival and lower rates of end-stage kidney disease. Here we describe a patient with MPA ANCA who presented with diffuse alveolar hemorrhage and, six months later, recurrent pulmonary hemorrhage with renal sparing while off therapy.

## Introduction

Anti-neutrophil cytoplasmic antibody (ANCA)-associated vasculitis (AAV) is a group of disorders that includes microscopic polyangiitis (MPA), granulomatosis with polyangiitis (GPA), and eosinophilic GPA (EGPA; alternatively, Churg-Strauss syndrome). Each AAV subtype involves severe, systemic, small-vessel vasculitis. ANCA vasculitis has an estimated annual incidence of 20 per million [1]. During the last three decades, a steady increase in the incidence and prevalence of AAV has been reported globally, potentially due to the evolution and proliferation of new diagnostic criteria, widespread use of ANCA serology, and increased health provider awareness of these disorders [2]. The overall annual incidence rate of AAV in children is estimated to be 3.2 per million [2]. There is a clear increase in the age-specific incidence of AAV. The male-to-female incidence ratio is variable [2].

AAV is characterized by the development of autoantibodies to the neutrophil proteins leukocyte proteinase 3 (PR3-ANCA) or myeloperoxidase (MPO-ANCA). The most common ANCA type associated with GPA is PR3, while the most common type associated with MPA is MPO. AAV pathogenesis involves excessive activation of neutrophils, which subsequently release inflammatory cytokines, reactive oxygen species, and lytic enzymes. In addition, this aberrant neutrophil activation induces excessive formation of neutrophil extracellular traps (NETs), which are harmful to small vessels. Excess NETs are also involved in the production of ANCAs, causing a vicious cycle of NET formation and ANCA production, which is involved in the pathogenesis of AAV [3].

MPA and GPA share substantial clinical and pathologic overlap in many of their clinical features, making it difficult to differentiate between the two disorders without biopsy results. GPA usually starts as a granulomatous disease of the respiratory tract. It progresses to systemic disease with PR3-ANCA-associated vasculitis in many patients [4], suggesting an aberrant cell-mediated immune response to exogenous or endogenous antigens in the respiratory tract. GPA results in granuloma formation, which putatively represents the lymphoid structures ultimately responsible for PR3-ANCA production [5]. MPA primarily involves the kidneys and respiratory tract, manifesting as necrotizing glomerulonephritis (GN) and pulmonary vasculitis. MPO-ANCA is implicated in the pathogenesis of this condition; however, not all MPA cases are positive for MPO-ANCA [6].

The diagnosis of AAV rests on suggestive clinical features, ANCA testing and biopsy results of target organs. ANCA is positive in 80% to 90% of patients with either MPA or GPA [6], which is helpful in establishing a diagnosis. However, a negative ANCA test does not exclude AAV because 10% to 20% of patients are ANCA-negative at the time of diagnosis [6]. Tissue biopsy on many instances provides the data necessary to establish a diagnosis of AAV. Findings from kidney biopsies in patients with GN range from focal GN to segmental GN to diffuse necrotizing and crescentic GN in patients with acute kidney injury. Typically, deposits have been described as pauci-immune depositions in a focal or segmental GN pattern [7-10].

## Case presentation

A 71-year-old female patient with a medical history of hypertension, coronary artery disease, hyperlipidemia, and diastolic congestive heart failure presented to the hospital complaining of severe headache and chest pain. She reported the headache was persistent for several weeks, unilateral to the right side, severe and pressure-like in nature, and worsened with any local pressure in the area of the headache. She reported trying over-the-counter Tylenol and ibuprofen without relief. She denied associated vision changes, nausea/vomiting, fever, or loss of consciousness. She endorsed dyspnea that worsened with exertion for several hours before the presentation but denied orthopnea, pedal edema, cough, hemoptysis, or fever. A review of the systems was positive for fatigue, knee pain, and generalized weakness.

On presentation, she was afebrile with a heart rate of 89 beats per minute, a respiratory rate of 18 breaths per minute, a blood pressure of 165/80 mm Hg, and an oxygen saturation of 99% on 2 L of O_2_. Physical examination showed no visible respiratory distress while on O_2_ by nasal cannula, normal exam of the head and oral cavity, supple neck, normal respiratory effort and no added sounds, normal heart sounds with no murmurs or gallops, no clubbing, soft and non-tender abdomen with normoactive bowel sounds, and 1+ edema in the lower extremities at the ankles bilaterally. Labs were significant for the following: white blood cell (WBC) count of 13.9 x 10^9^/L (predominantly neutrophils), eosinophil absolute count of 300 cells/mL, hemoglobin level of 9.4 g/dL, mean corpuscular volume of 87 fl, platelet count of 512 x 10^9^/L, and reticulocyte percentage of 1.2%. A basic metabolic panel, liver function tests, and transaminases were within normal limits. Her erythrocyte sedimentation rate was >130 mm/hr, and her C-reactive protein level was 189 mg/L. Urinalysis showed trace protein and small hyaline casts but was negative for WBCs and red blood cells. COVID-19 PCR and antibody tests were negative.

**Figure 1:**
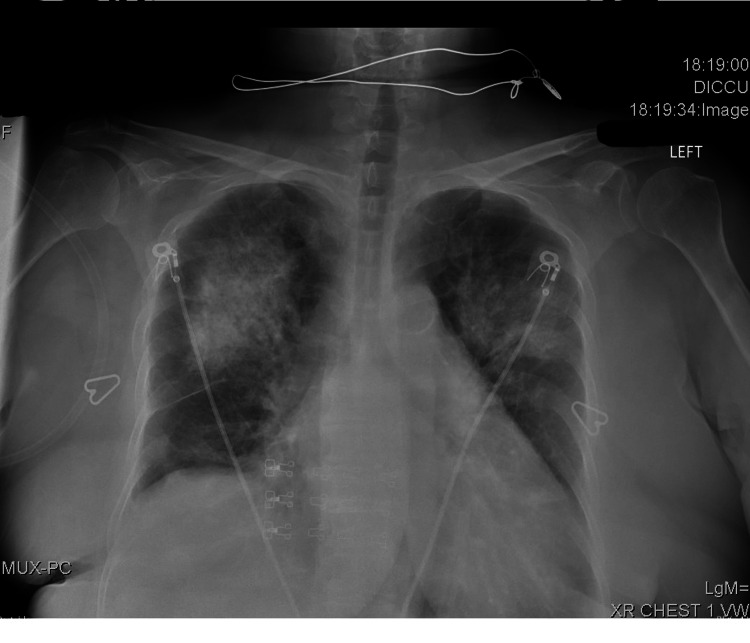
Chest x-ray (anteroposterior view) showing bilateral dense pulmonary infiltrates, air bronchograms, and fluid in the interlobar fissure on the right side.

**Figure 2:**
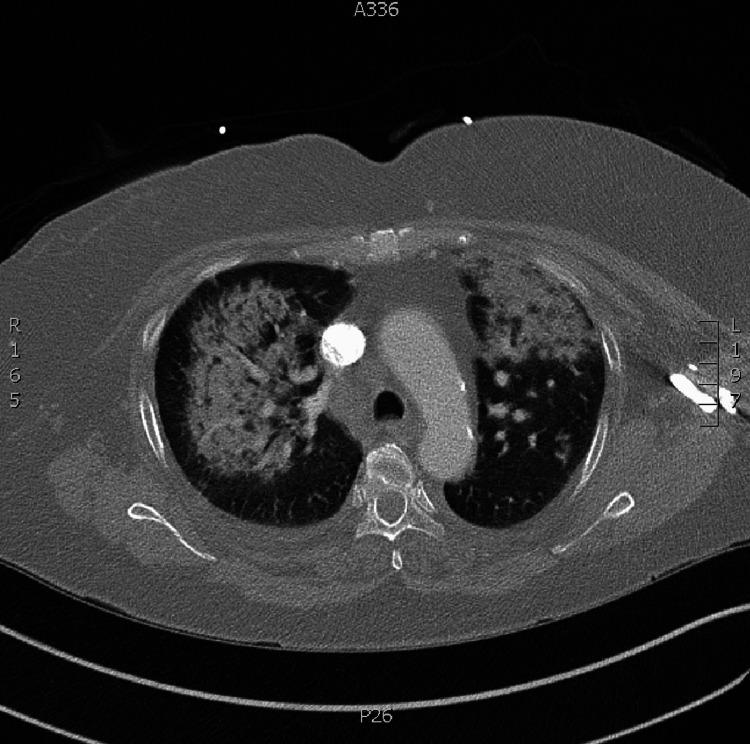
Chest computed tomography scan (lung window) during the primary admission showing bilateral patchy pulmonary infiltrates/consolidations, air bronchograms, bilateral ground glass opacities, and a small right-sided pleural effusion.

An anteroposterior chest x-ray on admission showed bilateral pulmonary infiltrates with air bronchograms (Figure 1). A computed tomography (CT) angiography of the chest showed no evidence for a pulmonary embolism, but did show bilateral patchy pulmonary infiltrates, ground glass opacities, and a small right pleural effusion (Figure 2).

After admission, the patient was started on IV antibiotics with a working diagnosis of bilateral multi-lobar pneumonia. Blood cultures, methicillin-resistant *Staphylococcus aureus* nasal swabs, and urine Legionella antigens were negative. Due to an initial suspicion for giant cell arteritis, the patient was also started on oral prednisone therapy at 1 mg/kg, and later, a temporal artery biopsy was pursued. A rheumatologic workup was positive for perinuclear ANCA (titer 1:1280) and MPO-ANCA (34 AU/mL), but negative for anti-PR3 (15 AU/mL) and anti-glomerular basement membrane; complement levels were normal. A hepatitis panel was negative.

**Figure 3:**
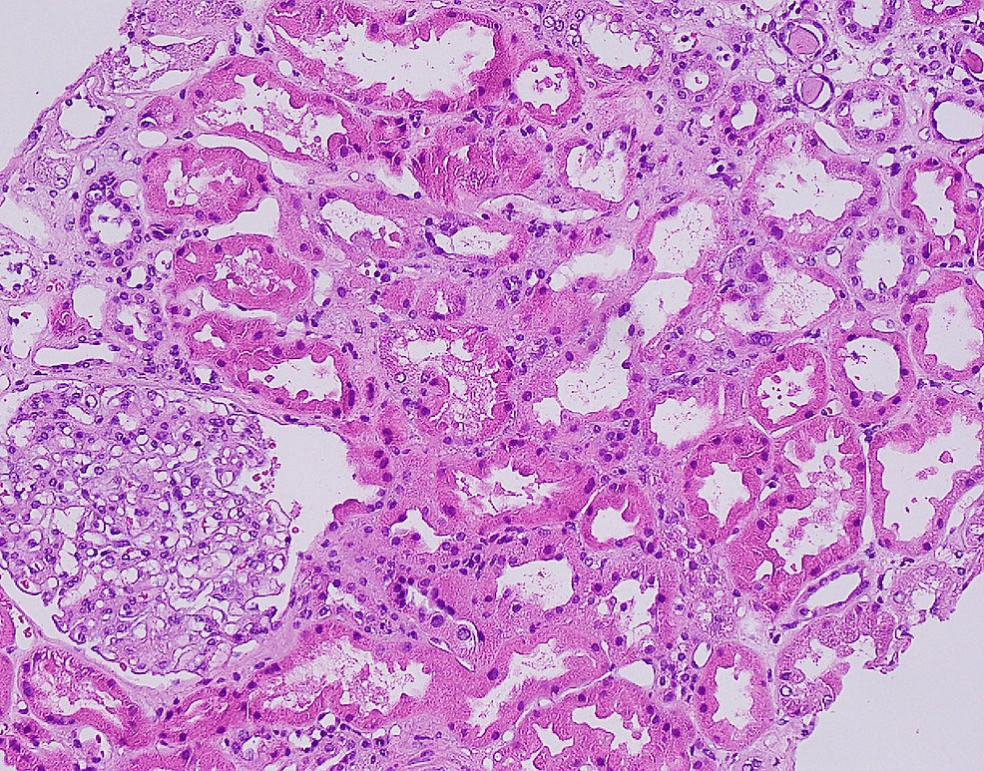
Hematoxylin and eosin (HE) shows globally sclerotic glomeruli and interstitial fibrosis.

**Figure 4:**
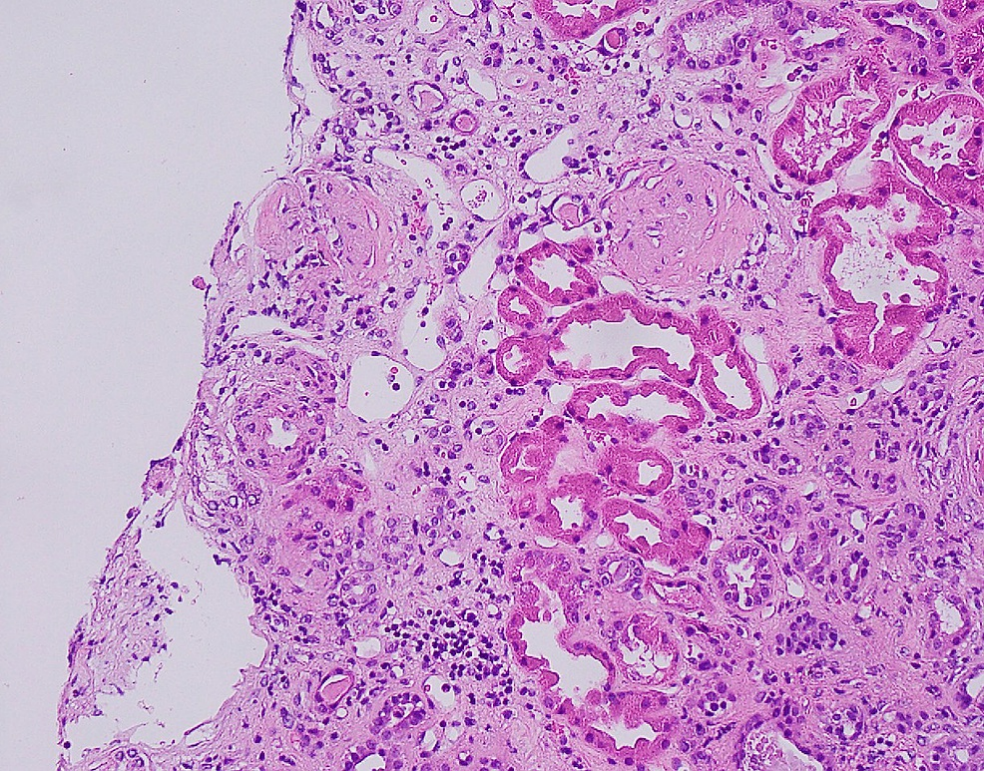
Hematoxylin and eosin (HE) stain shows ATN. ATN: acute tubular necrosis.

**Figure 5:**
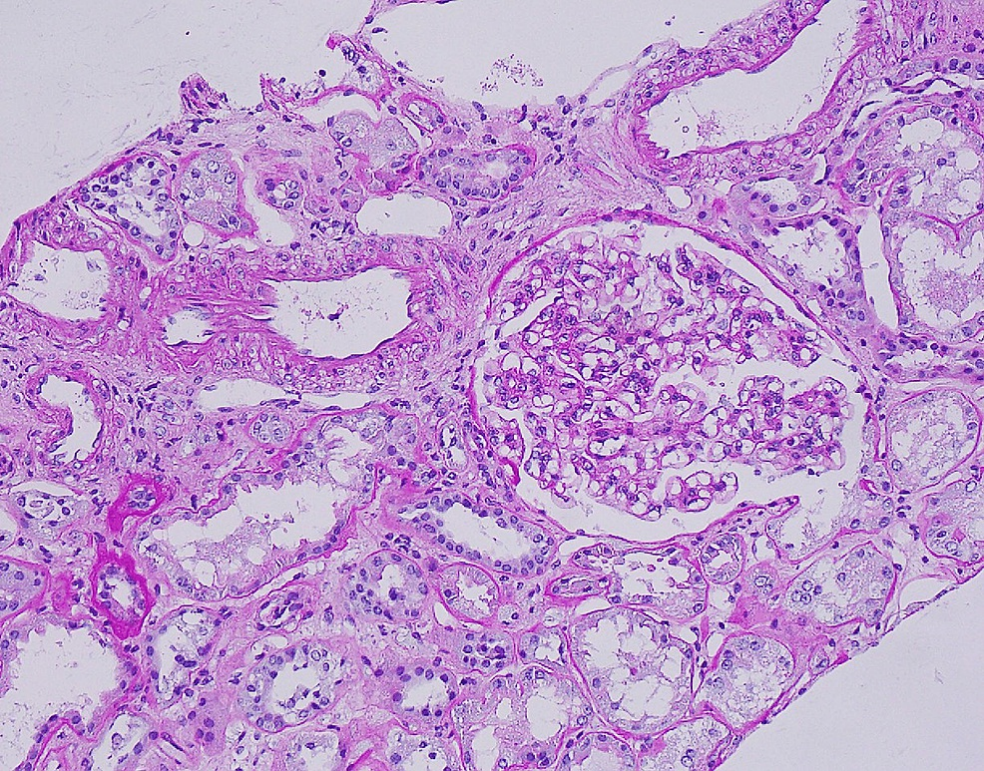
Periodic acid-Schiff (PAS) stain showing a normal glomerulus.

A temporal artery biopsy showed no evidence of inflammation or granulomas. A kidney biopsy was obtained after consulting with the nephrology service due to the strongly positive ANCA titer and high inflammatory marker levels. The biopsy, which contained 17 glomeruli, showed global sclerosis in 9 glomeruli; the non-sclerotic glomeruli were histologically unremarkable. The tubulointerstitium demonstrated mild interstitial fibrosis and tubular atrophy in 20% of the sample (Figures 3-5). Electron microscopy showed no electron-dense immune-type deposits or organized deposits. All immunofluorescence stains were negative in the glomeruli; there was no significant extraglomerular staining present, and there was no evidence of crescentic or necrotizing GN.

After discussing with nephrology, the patient was started on high-dose IV methylprednisolone at 500 mg daily for three days and was given a dose of IV rituximab for MPO AAV. Her oxygenation remained stable, and she was later weaned off oxygen. She was discharged with instructions to follow up with rheumatology for completion of induction therapy with rituximab and later initiation of maintenance therapy.

**Figure 6:**
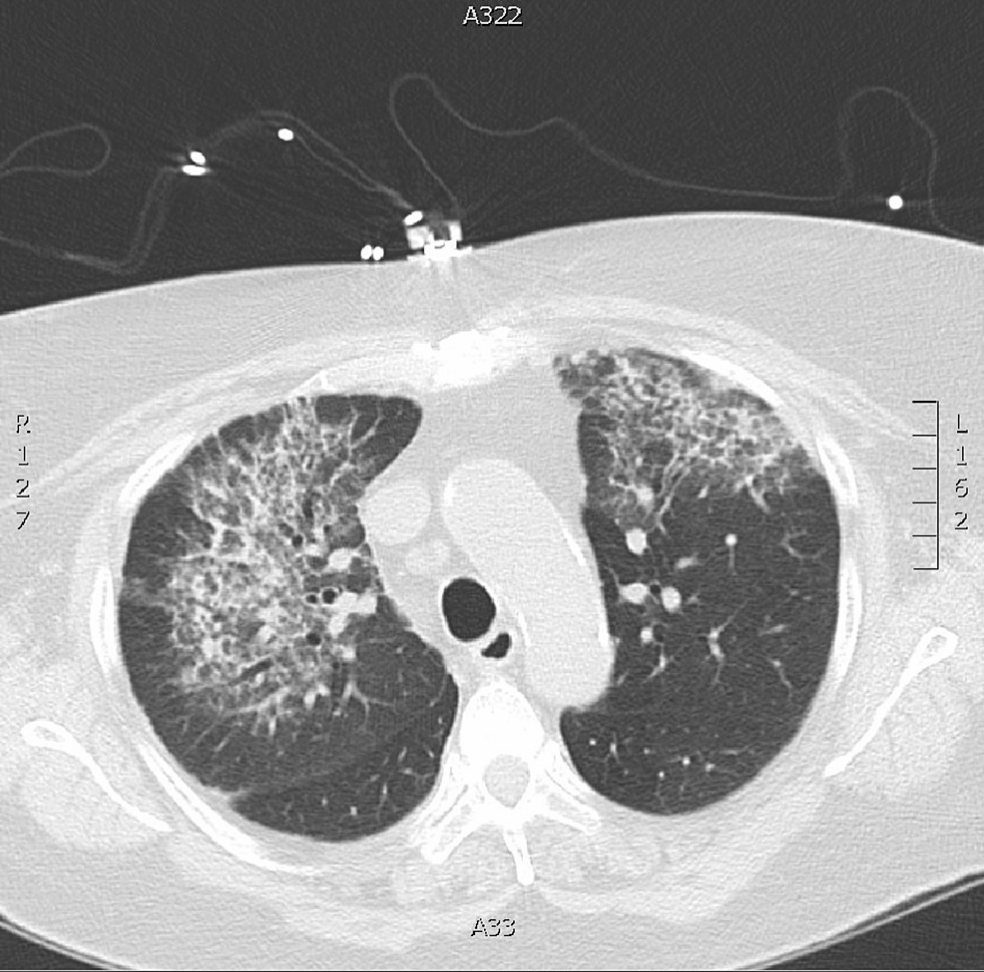
Chest computed tomography scan (lung window) at eight months post-original discharge showing bilateral infiltrates and ground glass.

The patient decided against further rituximab infusions and chose not to follow up with rheumatology. She presented to the hospital eight months later with severe dyspnea, chest pain, and hemoptysis. She was in acute hypoxemic respiratory failure and required 10 L of O_2_ by nasal cannula. Her inflammatory marker levels and ANCA titer were noted to be elevated again on this admission, and a chest CT scan again demonstrated dense bilateral pulmonary infiltrates and ground glass consistent with diffuse alveolar hemorrhage (Figure 6). She responded to high-dose IV steroid therapy, and her oxygenation improved before discharge. No further immunosuppression was started on the patient on this admission.

## Discussion

In the case of presentation with isolated pulmonary hemorrhage, consideration should be made for infectious etiologies. Bilateral pulmonary disease, lack of response to antimicrobials, and strong evidence for alternative diagnoses helped divert the diagnostic effort to alternative non-infectious etiologies. A review of previous medications is key in the evaluation of any pulmonary hemorrhage since anticoagulants and other medications associated with hypersensitivity reactions can induce pulmonary hemorrhage. Evidence to support a diagnosis of an alternate disorder as anti-glomerular basement membrane disease (anti-GBM) was lacking, including a normal titer of anti-GBM antibody and a normal kidney biopsy lacking Ig deposits. Diffuse mixed cryoglobulinemia was also a consideration; however, rheumatoid factor and hepatitis serologies were negative, making this unlikely. 

AAV is a multisystem disorder that involves multiple organs. The most common targets for involvement are the kidneys and lower respiratory tract. GPA almost invariably involves the upper respiratory tract and can involve other organs, including the nervous system, gastrointestinal (GI) tract, and eyes, including the retina. Dermatologic involvement is more frequent with MPA than GPA, and pulmonary involvement in MPA is common. The diagnosis of AAV should be considered in subjects with suggestive symptoms associated with clinical evidence of involvement in the target organs mentioned above, including the respiratory tract, the presence of glomerulonephritis, or the presence of an otherwise unexplained neuropathy. On occasion, the manifestations of the disease might be limited to a single organ, as in the case of renal-limited disease or the reported cases of isolated pulmonary findings without other organ involvement in MPA patients [11]. A Japanese prospective cohort study found that over 90% of high-resolution CT scans were abnormal in MPA patients [12]. The most common findings on imaging included ground glass opacities (50%), reticulation (48%), traction bronchiectasis (42%), honeycombing (31%), and emphysema (22%) [10]. Another study found that the most common pulmonary patterns in AAV patients were usual interstitial pneumonia, fibrotic nonspecific interstitial pneumonia, and combined pulmonary fibrosis and emphysema patterns [11].

Since the treatment of pulmonary hemorrhage includes the administration of high-dose glucocorticoids, it is recommended that a careful assessment for infectious etiologies must be carried out before initiating systemic glucocorticoids or immunosuppressive treatments. In many cases, primary antimicrobial therapy is often initiated while waiting for the results of the infectious workup, as in the case presented here. 

Left untreated, GPA and MPA can carry a two-year mortality rate of up to 90% [13]. Even with treatment, these disorders still carry a 2.7-fold increased risk of death (compared to the general population), which is mostly driven by infections and ongoing vasculitis in the first year after diagnosis [14]. Currently, the risk of end-stage renal disease, remission rates, and death has substantially decreased with the advances in treatment and use of cyclophosphamide and rituximab for the treatment of MPA and GPA; however, the risk of relapse has not changed and remains high [13,15]. Multiple independent risk factors for death have been identified, including age >65 years, creatinine level >1.47 mg/dL, severe GI involvement, and mononeuritis multiplex [15].

## Conclusions

Involvement of the respiratory system is a very common and important feature of AAV, especially for MPA and GPA. Isolated pulmonary involvement in these disorders does occur, and this might be the first or only target organ involved in AAV without associated GN. There is substantial overlap in many of the clinical pulmonary features of AAV. Patients treated for AAV have improved rates of remission and survival, but rates of relapse continue to be unaffected by the current treatment protocols.
